# Airflow Modeling for Citrus under Protective Screens

**DOI:** 10.3390/s24196200

**Published:** 2024-09-25

**Authors:** Liubov Kurafeeva, Rich Wolski, Chandra Krintz, Thomas Smyth

**Affiliations:** 1 Computer Science Department, University of California, Santa Barbara, CA 93106, USA; 2 Geography and Environmental Science Department, Liverpool Hope University, Liverpool L16 9JD, UK

**Keywords:** controlled environment agriculture, wind modeling, CFD, validation, citrus crop

## Abstract

This study explores the development and validation of an airflow model to support climate prediction for Citrus Under Protective Screens (CUPS) in California. CUPS is a permeable screen structure designed to protect a field of citrus trees from large insects including the vector that causes the devastating citrus greening disease. Because screen structures modify the environmental conditions (e.g., temperature, relative humidity, airflow), farm management and treatment strategies (e.g., pesticide spraying events) must be modified to account for these differences. Toward this end, we develop a model for predicting wind speed and direction in a commercial-scale research CUPS, using a computational fluid dynamics (CFD) model. We describe the model and validate it in two ways. In the first, we model a small-scale replica CUPS under controlled conditions and compare modeled and measured airflow in and around the replica structure. In the second, we model the full-scale CUPS and use historical measurements to “back test” the model’s accuracy. In both settings, the modeled airflow values fall within statistical confidence intervals generated from the corresponding measurements of the conditions being modeled. These findings suggest that the model can aid decision support and smart agriculture solutions for farmers as they adapt their farm management practices for CUPS structures.

## 1. Introduction

Citrus is a crop of substantial economic importance to the US agriculture industry. In the 2022–2023 season, it was valued by the USDA at $2.59 billion [1]. Florida, Texas, Arizona, and California are the primary US producers of citrus, with California accounting for 79% of US production during this period [1]. Once a leader in citrus production in the US, Florida’s citrus production has declined by 92% since the 2003–2004 season (producing only 17% of US production in 2022–2023).

A key contributor to the decline in citrus production is citrus greening disease, also known as Huanglongbing (HLB) [2,3]. HLB is a devastating disease without an approved remediation that is spread by an insect called the Asian Citrus Psyllid (ACP) and which leads to premature fruit drop, fruit that never ripens, and eventual tree death. HLB infection is responsible for the loss of more than $4.5 billion in revenue by the US citrus industry over the period 2006 to 2010 [4]. In California, where HLB infection has not yet reached epidemic proportions, it has been found recently in multiple counties primarily in southern California [5,6]. The concern is that it will migrate to, and become endemic in, California’s central valley where much of California’s (and US) commercial citrus is produced.

One way to protect citrus trees from ACP and HLB infection is to grow the trees under field-scale, protective screens to create a physical barrier between the insects and the plants. Such structures are called Citrus Under Protective Screen (CUPS) [7]. The use of CUPS has been the subject of much research since the early 2020s [7,8,9]. Today CUPS is estimated to cost $0.69 to $1.03 per square foot ($30,000 to $45,000 per acre) and has been shown to be more effective than other methods for HLB protection in Florida [7,9,10]. Screen and net houses are also used to exclude pests and to provide protection from the sun and wind for a variety of crops in the US and worldwide [11]. Moreover, citrus groves in Florida have been shown to respond positively to the buffered environmental conditions of CUPS, yielding improved fruit quantity and quality [10]. However, CUPS has yet to be studied for HLB protection in California where growing conditions and crop varieties differ substantially from those in Florida.

Our research investigates the use of CUPS in California using the first, and currently only, commercial-scale (4-acre) research CUPS located at the Lindcove Research Extension Center (LREC) [12] in the central valley of California. The LREC CUPS is instrumented with a large-scale Internet-of-Things (IoT) deployment that consists of wireless communications, resource-constrained (battery-powered) edge computers, cameras, and multiple weather stations equipped with wind sensors. Our goal is to use this system to develop modeling, prediction, and data-driven actuation and control systems that help growers adapt their agricultural practices for CUPS deployments. Generally, our work focuses on automation and digital systems to monitor and manage the environmental growing conditions within a CUPS. Such research is important to the agriculture industry because screen structures modify the environmental conditions (e.g., shading, temperature, relative humidity, airflow) in ways that affect plant health and the growing cycle.

In this paper, we focus specifically on airflow within the CUPS for three reasons. The first is that airflow ((i.e., wind) can have a dramatic effect on other environmental factors (e.g., water evaporation). Secondly, unlike relative humidity or temperature, airflow is dynamically changing and its effects are often localized due to turbulence caused by obstacles and topological features in and around the growing area. Thirdly, understanding and predicting airflow is critical to managing important farming practices such as irrigation, the application of sprayed inputs, and sprayed treatment strategies (e.g., pesticides, fertilizers, sprayed water for frost prevention, etc.).

Note that we treat the commercial-scale CUPS facility at LREC as a living laboratory. That is, our work does not evaluate the efficiency of CUPS from the biological and agricultural perspective. Instead, it assumes that CUPS can, and will, play an increasingly important role in the future of citrus production and our research shows how IoT can be used to support this role from a climate control and prediction viewpoint.

Since the CUPS is neither a climate-controlled structure nor fully exposed to the weather, its porous screen walls, roof, and internal structural elements create complex airflow patterns that are difficult to measure and predict. Our research explores whether it is possible to accurately model and predict wind flow patterns inside the structure using external measurements.

Specifically, we present a model for airflow through and around the CUPS using computational fluid dynamics (CFD). CFD models provide accurate representations and predictive capabilities in a variety of engineering and environmental disciplines [13,14,15] including mesoscale weather forecasting [16,17]. Our CFD model consists of Navier–Stokes equations and uses a Darcy–Forchheimer model to simulate conditions inside and outside the CUPS (we use OpenFOAM [18] to perform the calculations).

In the sections that follow, we describe the model and validate it in two settings: a scaled-down physical model of the CUPS sited in a controlled, miniaturized IoT environment, and in the field, using the commercial-scale CUPS facility at LREC. We compare our model predictions against empirical measurements. To validate our model at a commercial scale, where there are uncontrolled external weather conditions that could affect model accuracy, we perform back-testing using historical data from weather station sensors that are deployed inside and outside of the LREC CUPS. In both settings the modeled airflow values fall within statistical confidence intervals generated from the corresponding measurements of the conditions being modeled. That is, in both a controlled experiment and in the field, the CFD model generates simulated values that are consistent with the measurement uncertainty associated with the sensors that are used to gather the comparative measurements.

These findings suggest that our model can be used as part of decision-support tools that aid farmers in adapting their agricultural practices for CUPS deployments. For example, current practice is to make crop management decisions using a combination of sensor measurements covering many square kilometers (e.g. nearby airport meteorological data), possibly with a single local weather station. The validation presented in this paper shows model accuracy at the meter scale within the CUPS which should lead to more accurate decision support and actuation. In this way, our work lays the foundation for new intelligent actuation and control systems for spraying activities, irrigation scheduling, and frost prevention under CUPS.

## 2. Materials and Methods

In this section, we formalize our research task and detail the methods that we used to accomplish it. Specifically, we describe the requirements that we attempted to satisfy (Section 2.5) and discuss related efforts that inform our approach (Section 2.3). We also overview the CUPS IoT infrastructure, the sensor data we collected (Section 2.4), the physics model (Section 2.5.1), and the software (Section 2.5.2) that was used in this study. Finally, in Section 2.5.3, we describe our deployment and plans for using this model as part of an IoT decision support system for growers.

### 2.1. CUPS Physical Infrastructure and Sensing Equipment

**Figure 1:**
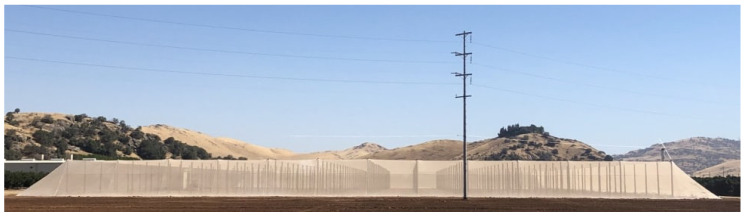
CUPS structure side view.

**Figure 2:**
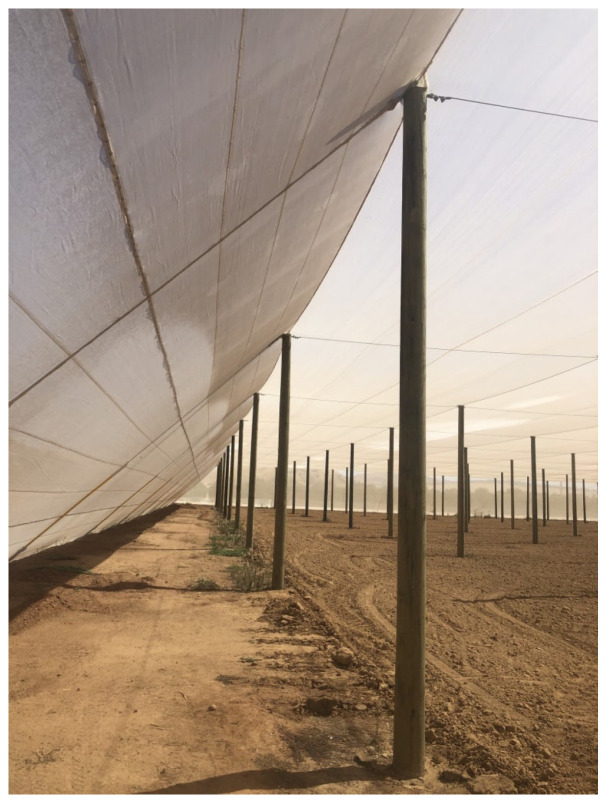
LREC CUPS edge and screen close-up photo.

Citrus Under Protective Screen (or CUPS) is a rectangular, field-scale, porous structure for growing citrus trees. The distinct feature of this structure is a regular screen covering it on four sides and the roof. The overall view of this structure is depicted in Figure 1 with a close-up view of the edge of the screen in Figure 2. The screen excludes the admission of insects larger than the screen size but admits attenuated sunlight and ventilation. For HLB prevention, the screen size should be no larger than 40 microns to prevent Asian Citrus Psyllid infestation [19].

The commercial-scale research structure located at LREC is approximately 5.5 m tall, 100 m wide, and 190 m long. The roof of the structure is supported by poles in a grid approximately 4.8 m and 8.5 m apart. Trees inside are planted every 2.4×4.3 m (for a total capacity of 32 trees per row and 390 trees per acre). This research began before trees were introduced as part of an effort by growers to understand the growing conditions within the structure. As such, our data, model, and validation reflect the pre-tree period of the study. We plan to validate our model further with young and mature trees as part of future work.

**Figure 3:**
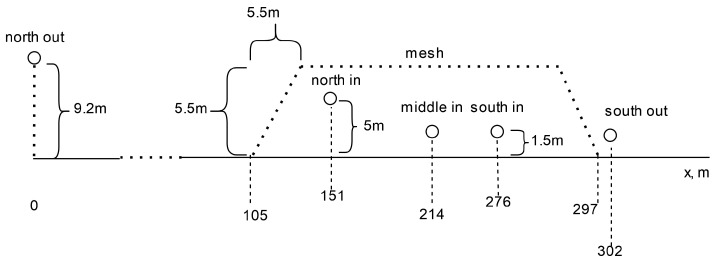
CUPS structure and sensor positioning.

We collected sensor data on the climate conditions inside the CUPS using AcuRite 01075RM 5-in-1 weather stations [20] and Davis Instruments Vantage Pro 2 weather stations [21], both equipped with cup anemometers. AcuRite is a brand from Chaney Instrument Company which is headquartered in Lake Geneva, WI USA. Davis Instruments is headquartered in Hayward, CA USA. The weather stations are part of another IoT research project in the CUPS. Thus, we leveraged the sensors and deployment infrastructure without introducing changes that may impact other projects (and increase costs). The weather stations that we used in this study were located in five locations (two outside the structure and three inside) as shown in Figure 3.

The *north out* location was located approximately 100 m north, outside of the north end of the CUPS. The anemometer at this location was mounted at a height of 10 m. The *north in* location was located 160 m due south of *north out* (60 m inside the northern boundary of the CUPS). The anemometer at this location was mounted at a height of 5 m. The *middle in* location was 214 m due south of *north out* (114 m inside the northern CUPS boundary). The anemometer at this location was mounted at a height of 1.5 m. The *south in* location was 276 m south of *north out* (176 m inside the northern CUPS boundary). The anemometer was mounted at 1.5 m. Finally, *south out* was located in 302 m south of *north out*, immediately outside the southernmost boundary of the CUPS, at a height of 1.5 m. While the sensors were oriented along the north–south axis of the CUPS, they were not aligned perfectly due to structure requirements dictated by the agricultural layout.

### 2.2. Formalization of the Task

The specific research task we pursue with this effort is *to develop and validate a CFD model for the CUPS that can be parameterized by local atmospheric measurement values (from IoT sensors) to produce a representation of airflow throughout the structure and to describe the accuracy associated with this validation.*

We focus on model validation, as opposed to specific use cases, because we believe that an accurate model of airflow is foundational to multiple applications including frost prevention, spraying activities, irrigation scheduling, and CUPS maintenance. Further, we wish to understand whether relatively inexpensive meteorological sensors can be used at a commercial scale or whether more expensive (and more precise) sensors are necessary. We validate only horizontal wind speed because the weather stations to which we had access for this study are both inexpensive enough for an agricultural deployment, and designed for long-lived outdoor use. As a result, they only support cup anemometers.

We employed two separate validation methodologies. In the first, we constructed a scaled-down physical replica of the CUPS which we instrumented with small-scale anemometers that were more accurate than the weather station anemometers. We developed, tested, and calibrated the CFD model under controlled indoor conditions using fans to generate simulated wind (Section 2.6). Note that the results presented in Section 3.1 are for the model that generates results within the statistical uncertainty bounds of the anemometers used in the controlled experiments. We made heavy use of the controlled setting to explore different models with different hyperparameter settings that were not as accurate. In the second, we back-tested the model identified in the controlled environment using historical wind measurements taken from the full-scale CUPS structure (Section 3.2).

### 2.3. Related Work

The specific task of climate prediction for CUPS structures in California is an open question because the design and use of CUPS screen technology for citrus is in its infancy. Indeed, our team has access to the first (and currently only) commercial-scale CUPS research structure in California at the Lindcove Research and Extension Center (LREC). Existing research on CUPS is focused on productivity improvements enabled by CUPS, structure design, and on the return-on-investment enabled by CUPS in Florida [7,8,9]. Our work is distinct because it creates a digital model of meteorological conditions (specifically airflow) within a commercial-scale CUPS located in California’s Central Valley.

Other work has successfully employed various types of modeling to support agricultural practices [22]. Many target optimizing resource use and costs [23,24]. Still, other related work advances the state of the art in IoT platforms [25], unmanned vehicles [26,27], sensing techniques [28], and management systems [29]. Most existing agricultural systems target specific crops, growing environments, and tasks.

More closely related works by Adamides et al. [30] and Park et al. [31] discuss in-depth options for data collecting for climate prediction for use by growers. These studies focus on open fields (without structures/enclosures) that are visible from satellite imagery and relatively cheap to instrument using solar-powered weather stations. These efforts do not mention the instruments and mechanics to analyze the data and either rely on expert human labor or omit model details.

More generally, wind speed and climate prediction outside of agricultural sector applications are popular research tasks and can be categorized by the spatial scale over which the predictions are made. Global climate and wind speed prediction, like Slingo et al. [16] concentrate on city, state, and larger aggregated areas. Smaller scale, outdoor wind monitoring, and modeling have been the focus of wind farm settings [32,33,34].

Lawan et al. [17] provides a comprehensive review of different local point wind-speed prediction models and divides them according to the timing between known data and required prediction; however, none of the cited works take into account the wind map of the local area. A recent example of a comparison of different models for this task was described by Li et al. [35]. They consider AI and machine learning approaches for wind speed prediction. Works that are more closely related to ours describe air movements in enclosed areas such as enclosed spaces (rooms). Researchers model these settings using computational fluid dynamics, e.g., [15]. These approaches grew in popularity during the COVID-19 pandemic, where they were used for understanding human safety in these settings. Examples of successful models can be found in the review by Mohmadi et al. [36]. Works like Bhattacharyya et al. [37] and others [14,38] also inspire our work on wind and airflow predictions. Most of those works use CFD models as we do. However, they focus on slow air movement (not wind speed) and work with enclosed (non-porous) areas with limited inputs and outputs (like vents, windows, and doors). The methods applied in those works influence our approach but cannot be applied directly given the porosity and scale of the CUPS.

Other related work attempts to model tree and wind interaction. Schindler et al. [39], for example, discusses the effects of different wind speed ranges on trees. Endalew et al. [40] validates the wind and tree canopy interaction using similar CFD methods mentioned above, but in a strictly controlled environment which allows their model to use solvers that are too slow for commercial-scale agriculture and only capable of small-scale computation. There are also other works [41,42] that employ CFD-related approaches. These all use different solvers, options, and turbulence properties from those we found to be useful for commercial-scale CUPS. Others use different core calculation parameters that are not appropriate for the setting we target. Although not directly applicable, these approaches informed our model exploration.

Lastly, other work, such as Huang et al. [43], model wind speed and air movement interacting with large solid objects (e.g., buildings). The goal of these works is to improve the architectural design of buildings and streets by addressing climate, air quality, and noise issues. Another example of such work is [44] which considers building geometry and Cao et al. [13] which considers ventilation performance. These approaches are larger scale than other related work on modeling wind flow patterns using CFD approaches but their models are less accurate and less detailed compared to our approach for the settings we target. To our knowledge, ours is the first research that focuses on modeling wind flow patterns inside CUPS.

### 2.4. Historical Data Description

For back-testing validation, we employ a historical archive of IoT sensor data from the LREC CUPS. The weather stations of the CUPS are connected to a co-located server (on-farm) via a wireless network. The server sends the data to campus cloud storage to which it is connected via the Internet. Each weather station is equipped with sensors for temperature, humidity, wind speed, wind direction, rain rate, UV level, and other characteristics. The weather station collects the data from the sensors and reports the records to the server every 5 min. This passive data collection has been ongoing since January 2022 and has approximately 151,000 records for each weather station. We filter the data to identify contiguous periods of measurement and to remove periods when one or more sensors were malfunctioning. For the purposes of this work, we extract wind speed and wind direction from the archive.

### 2.5. The CFD Model Requirements

We require that the CFD model accurately predicts wind motion (velocity and direction) within the CUPS structure given wind sensor values upwind from the structure (which we used as inputs to the model). Using such a model, it should be possible to predict internal wind flows based on localized measurements of wind conditions. The model should also be one that can be optimized (perhaps through the application of high-performance computing resources) so that it can generate these internal predictions in real-time or near real-time.

Many popular model choices for agricultural tasks [45] or wind prediction tasks [46] are AI-based solutions. The advantage of using a physics-based approach as opposed to one based strictly on machine learning is the ability to make an inference from relatively parsimonious data. In contrast, the use of AI typically requires very large amounts of data for training and establishment of initial values [47]. For the commercial scale CUPS, generating datasets of sufficient size and quality necessary for training appears, at this time, to be infeasible. We thus employ an approach that is physics-based and capable of predicting wind speed and direction across a farm-scale screen structure using more limited data that can be obtained in the commercial CUPS setting.

Our experience with IoT systems for agriculture and digital agriculture applications have led us to believe that complex use cases such as frost prevention, spray control, irrigation scheduling, etc. within the CUPS, require an airflow model that is capable of representing conditions in both low and high-pressure zones (e.g., screen fabric edges) and the turbulent flows that occur in these zones. Moreover, many of these applications require real-time or near-real-time inferences and predictions. Our ability to optimize the CFD model (possibly using large-scale computing resources such as the cloud) has also influenced our choice of model.

#### 2.5.1. Choice of Model

To address the challenges imposed by these requirements, we propose to use a physics-based computational model to model airflow under CUPS. Because our task involves simulation of the interaction of the wind inside the CUPS structure, we employ a physical model for fluid and gas interactions with other fluids and rigid bodies. The primary model used in our equations for fluid flow and heat transfer is the Navier–Stokes model (momentum equations), defined in Equation (1):

(1) $$\frac{\partial \rho }{\partial t}+\frac{\partial }{\partial x_{j}}\left[\rho u_{j}\right]=0$$

The main factor contributing to the complexity of our research task is the permeable nature of the screen. Because the screen has a complicated geometry, it is computationally infeasible without very large-scale computing resources to treat each screen element as a rigid body. The screen comprises polymer threads that are interwoven to create rectangular openings no larger than 40 microns on a side. In a commercial structure measuring 190 m by 100 m by 5.5 m, there are more than $1.4\times 10^{13}$ screen openings. This scale is too large to make modeling of each screen element computationally practical. To reduce simulation time, we treat the screen as a porous media [48]—a material containing voids through which fluid can flow, to reduce the computational cost of the model. This theoretical concept has been broadly used in industry [49,50] and research [51,52] shows that using such emulation structures that are penetrative by target liquid or gas but have rigid base are effective (and significantly more efficient) alternatives to rigid bodies.

We use the Darcy–Forchheimer equations for fluids through porous media [53] to model screen porosity.

(2) $$S_{i}=-\nu (D_{ij}+\frac{1}{2}\rho |u_{kk}\left|F_{ij}\right)u_{i}$$

The unknown factors for Darcy–Forchheimer are the screen description coefficients. We calculate the Darcy and Forchheimer coefficients using the geometrical calculation method from a screen sample that we obtained from the CUPS structure. To enable this, we take a high-resolution photo of the screen stretched over a dark sheet. From this image, using image processing algorithms, we calculate the relation between the holes and the material of the screen. We use this relation to estimate the coefficients as parameter $\phi =\frac{Open\;area\;of\;the\;perforated\;plate\;}{Total\;area\;of\;the\;perforated\;plate\;}$ The steps to calculate the Forchheimer coefficient *f* are performed using the following equations.

(3) $$0<\frac{L}{d_{h}}<0.015\;and\;Re>10^{5}$$

(4) $$k=\left[0.707\cdot {\left(1-\phi \right)}^{0.375}+1-\phi^{2}\right]\cdot \frac{1}{\phi^{2}}$$

(5) $$f=\frac{k}{L}$$

Another way to estimate the coefficients is to run a parameter sweep of possible coefficient values in a controlled setting and to choose the coefficients that correlate with the most accurate registration between measurements and model outputs. We discuss this approach more completely in Section 2.6.

#### 2.5.2. Choice of Software

The software we employ must accomplish three interacting tasks. It must generate a 3D representation of the solid and porous objects that comprise the CUPS. It must solve the physics equations that describe the airflow propagation through the structure. It must plot and render the results of the physics modeling for visualization purposes.

Blender: Modeling the Structure in 3D. We first create a full 3D model of the CUPS structure that captures the required properties and allows us to apply numerical methods and simulations to the model. Using Blender [54], we created a model 3D model of the structure, following the guidelines and measurements that growers provided. The model consists of a plane that represents ground in the area, a grid of poles in the shape of cylinders, and 5 planes to represent different screen sections. All parts were imported separately in STL format to apply specific interaction rules for OpenFOAM [18] use.

OpenFOAM: Meshing and calculation. Next, we discretize the 3D model onto a grid of cells. The step involves running multiple distinct algorithms to build the environment around the structure, importing each of the 3D model components (grid of poles and screen sections), and applying their properties such as materials and their parameters. Discretization is an essential part of the processing because all of the discussed models calculate changes cell by cell, which means that the number of calculations required grows with the number of cells, but also increases the accuracy of predictions. We found the effective trade-off between speed and precision using the *surfaceFeatureExtract* [55] tool. The area around solid objects has 5 layers with a cell size of approximately 5 cm × 10 cm × 10 cm. The area surrounding the screen has 8–10 layers with a cell size of approximately 2 cm × 5 cm × 2 cm. The empty areas have the largest cell size of approximately 25 cm × 50 cm × 25 cm. The layers of different size cells are separated by 1–3 layers to make the transition gradual. This balance in large and small cells and the gradient in between helps to reduce the calculation time while maintaining the accuracy of the calculation when transitioning between areas.

To implement the CFD calculation using the cellular representation of the CUPS structure, we used the open source software OpenFOAM v2312 [18]. This software includes numerous solvers (algorithms to calculate the spreading of the forces, and input conditions in the modeled environment) for fluids and rigid body interaction as well as ways to include the Darcy–Forchheimer model. OpenFOAM is also broadly used to study the fluid dynamics of wind in industry [14,44] and architecture [13] for safety testing. OpenFOAM requires that we select a primary solver that will determine the main paths of calculations and parameters that will be propagated, such as wind speed and pressure. It also takes as input the rules for these calculations and the addition of other, more specific solvers for enhanced model features (such as turbulence).

We use *porousSimpleFoam* [56] as the primary solver. This solver is steady-state (i.e., the calculations are expected to stabilize over time). The solver propagates the forces and changes, through the modeled space, until the most probable average conditions are achieved. The *porousSimpleFoam* is part of the Simple family of solvers included with OpenFOAM. However, this model does not include a turbulence calculation. We thus augment the primary solver with the k-epsilon Reynolds Averaged Simulation (RAS) method [57] to consider turbulence.

The overall algorithm is iterative. It discretizes time into steps that do not overlap. For the CFD computations in this study, we set this iteration interval to be 250 ms. This value was chosen experimentally. Larger values led to solver calculation failure (e.g., failure to converge) and smaller values significantly increased the overall computation time. For each iteration interval, the solver computes the new target parameters (e.g., wind speed, wind velocity, and pressure) for each cell in the spatial representation of the CUPS, based on the values in the cell and its neighboring cells at the previous iteration interval. The model iterates until values in each cell do not differ by more than a fixed threshold, after which time it is said to have “converged” to a steady state. Thus, for a set of input values, the model converges to a corresponding steady state for each cell in the modeled space.

The convergence threshold for the residual differences at each time step is a hyperparameter of the CFD model. However, degenerate spatial tessellations of cells can lead to premature and inaccurate convergence for some input values. For these reasons, we set the threshold to be very low and monitor the residuals. We terminate the CFD computation after 240 iterations. In all cases presented herein, the model reached convergence by this iteration interval. We also export the model state every 4 intervals to reduce the storage footprint required to analyze the model output. Once converged, the model output describes a set of atmospheric conditions (e.g., wind speed, wind velocity, temperature, etc.) at a centimeter scale ((i.e., in each cell) everywhere within the CUPS structure.

Data Processing. We use the *ParaView* [58] software package for plotting residuals, displaying and exporting model results, and performing other data visualizations. This software is compatible with the output format used by OpenFOAM so model data can be ingressed and manipulated directly without modification.

#### 2.5.3. Hardware

Our experimental setup consists of a Linux-based computer with Intel(R) Core(TM) i7-9700K CPU @ 3.60 GHz, 32 Gb RAM. In this initial study, and because we were validating the model accuracy (i.e., we did not have real-time deadlines), we used one core of the CPU. As a result, modeling the commercial-scale CUPS required approximately 7 h of wall-clock time to compute the model’s output. It is possible to improve this computational latency using shared-memory parallelism and/or a GPU implementation of the OpenFOAM solvers. Real-time or near-real-time response will likely require cloud-based high-performance computing resources. We plan to pursue these improvements as part of future work.

### 2.6. Modeling a Controlled Setting

**Figure 4:**
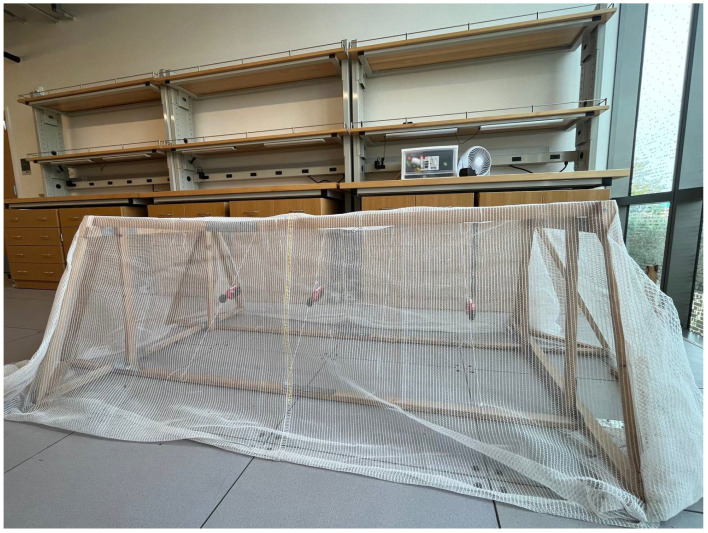
Scaled-down physical model of the LREC CUPS.

**Figure 5:**
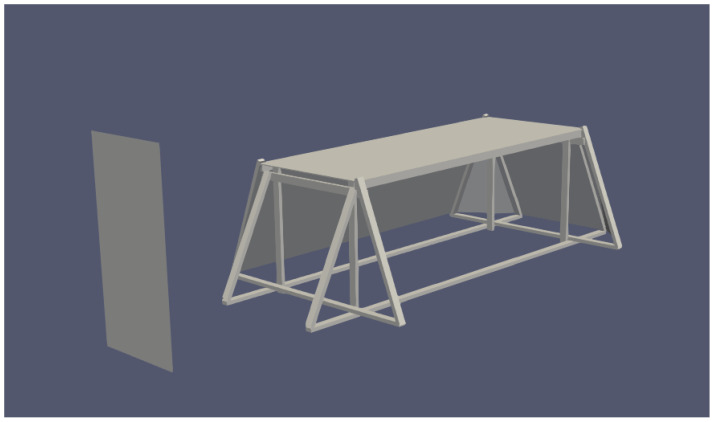
Depiction of the 3D model test set with a fan inlet (on the left) in Blender.

For model validation, we constructed a scaled-down “physical model” of the CUPS structure (shown in Figure 4). Figure 5 shows a digital rendering of this scaled test structure. This scaled version was also useful for the validation of the Darcy–Forchheimer coefficient estimations.

The scaled-down test structure used the same screen material as is installed on the commercial-scale CUPS. We placed the test structure in an indoor controlled setting (a storage area with climate control but no forced air) and instrumented it using anemometers placed at different locations inside and outside of the test structure.

#### CFD Model Development, Testing, and Calibration

The region of interest surrounding the test structure measures 244×122×67 cm, which is approximately 1/50,000th the scale of the the region we model surrounding the actual CUPS. We located two fans stacked vertically (used as a wind source) at a distance of 80 cm from one end of the test structure along its longest dimension perpendicular to the structure’s longest axis and placed the structure in the center of the test room for all experiments.

Note that the origin wind velocity (the wind velocity on the the surface of each fan blade) used to parameterize the CFD model is unknown and a specification that included maximum windspeed was unavailable for the fan models in this study (we used fans purchased at a local home-improvement store). Further, measuring the origin wind speed exactly at the fan blade surface is infeasible. Thus, we calibrated the measurements by starting the model with different origin wind speeds and comparing the average measured speed (after stabilization) 70 cm from the fan blades to the modeled average for that location. We tested the modeled origin wind speeds ranging from 4.5 m/s to 3.0 m/s, incrementing by 0.1 m/s and found that 3.2 m/s matched the average measured wind speed at 70 cm the most closely.

While both the test structure and the CUPS itself have angled side walls, we measured wind flow outside the structure (between the wind source and the windward edge) and within the maximal interior rectangular volume. This volume measured 170 cm × 63 cm × 63 cm within the interior of the test structure.

We instrumented the test structure using UNI-T UT363BT Mini LCD Digital Bluetooth Anemometers [59], equipped with a magnetic inductive wind speed sensor, which we used to measure wind speed at five locations oriented linearly along the centerline of the long axis. They were all suspended at a height of 30 cm from the floor.

**Figure 6:**
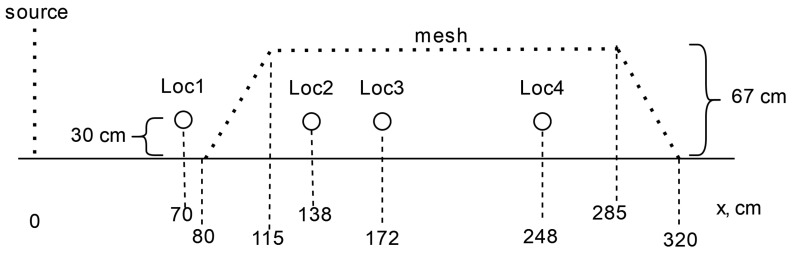
Calibration measurement locations relative to the test structure.

Figure 6 further details the placement configuration. Location 1 (Loc1) was 70 cm in front of the wind source and 10 cm in front of the leading edge of the test structure. Locations 2 through 4 (denoted Loc2, Loc3, and Loc4) were inside the structure at a distance of 138 cm, 172 cm, and 248 cm from the wind source, respectively.

## 3. Results

In this section, we describe our validation results. We first present the results for the scaled-down CUPS model in a controlled setting. We then present a validation study for the commercial-scale CUPS using back-testing.

### 3.1. Controlled Validation Results

We set the fans to a constant speed and took measurements within the scaled-down CUPS at each location every 5 s. Each experiment lasted 1000 s and we recorded measurements during the last 30 s of the experiment to give the conditions within the structure ample time to stabilize. This interval was determined empirically. During experimentation, we observed that a change in the fan speed required as much as 20 s before the measured values recentered to a new average speed. We chose 30 s to be certain of stability and verified that longer intervals after a change did not alter the standard deviation of the measurements substantively. We chose 1000 s of experimentation time fearing that conditions in the room outside the structure would need time to stabilize as well. Over the course of many validations, we did not observe variation caused by the length of the experimentation interval. We chose the last 30 s of the experimental period to be maximally conservative with respect to conditions stabilizing but shorter experimental intervals would likely produce a similar result. The room temperature during the experiments was 18 °C.

We modeled this structure using the OpenFOAM model (and the *porousSimpleFoam* solver) as described in Section 2.5.2. *porousSimpleFoam* is a steady-state solver, which means it returns average values once it converges. The model output is a gridded 3-dimensional rectangular volume of wind speed vectors. We used OpenFOAM’s adaptive mesh refinement feature [60] to generate the model grid that was eventually used to compute the wind velocities based on the internal geometry of the test structure. For validation purposes, we considered only the modeled values nearest the sensor locations.

**Figure 7:**
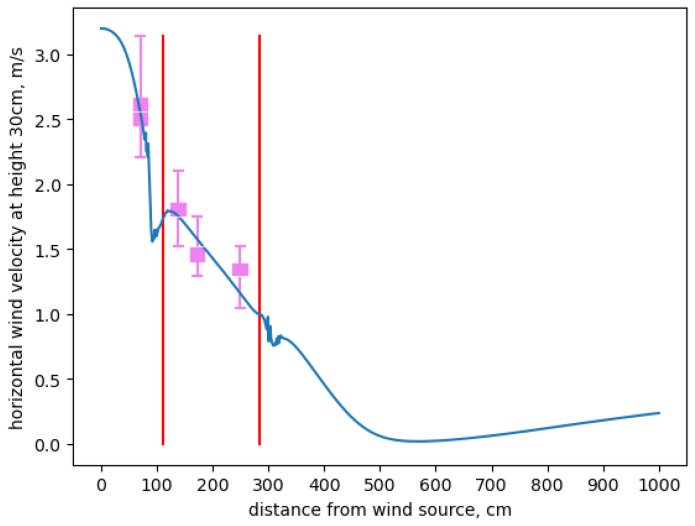
Controlled test structure model validation results. The blue line shows model prediction, the red lines depict the edges of the structure and the pink boxplots show sensor measurements.

Figure 7 shows the horizontal wind speed (on the *y*-axis) corresponding to the horizontal distance from the wind sources (along the *x*-axis) in centimeters. The vertical red lines indicate the boundaries of the maximal rectangular inner volume of the test structure. Data were taken from each sensor location over a 1000 second experiment period is summarized as a “box-and-whiskers” plot. The *x*-axis is the horizontal distance in centimeters from the wind source and *y*-axis is the wind speed in meters per second (m/s).

We depict the sensor measurement data for each location using “box-and-whisker” plots in which the vertical box boundaries show the upper and lower quartiles and the whiskers depict the extremum. The blue line in the figure shows the average horizontal wind speed computed by the OpenFOAM steady-state solver along the centerline of the test structure (i.e., through the sensor locations).

Table 1 further details these results. For each sensor location, column 2 shows the average measurement and standard deviation in meters per second, column 3 gives the corresponding modeled average value in meters per second and column 4 gives the two-sided 0.95 confidence interval on the mean from column 2. The modeled values for Loc1, Loc2, and Loc3 fall within the corresponding confidence intervals and Loc4 (shown in boldface) does not, but the measurement values recorded during the last 30 s of the experiment are all 1.29, making the confidence interval computation degenerate. If we substitute the manufacturer’s error interval of ±0.1 m/s for the sample standard deviation associated with measurement uncertainty, Loc4 would also be within statistical tolerance.

The lack of a sample standard deviation for Loc4 illustrates an important factor with respect to the practicality of the approach we validated. In this controlled setting, we chose small ruggedized anemometers with uncertainty intervals reported by their manufacturer that were substantially narrower than the error intervals for weather stations available in the full-scale CUPS. In neither setting (small-scale or full-scale) did the manufacturers report an uncertainty distribution for their anemometers nor did they indicate whether the interval (given as a ± value) corresponds to a specific α confidence level. Following best practices for standard uncertainty [61], we assumed that the measurement averages were normal with an unknown variance and used a Student’s *t*-statistic to compute confidence intervals. We chose α=0.05 as a standard confidence level [61]. For Loc4, we expected but did not observe measurements that exhibited sample variation. More precise laboratory-grade anemometers (to which we did not have access for this study) would allow for a more precise validation of Loc4. Thus, as it is, the validation is most properly construed as validating the CFD-modeled results against anemometers that are designed to be relatively inexpensive and deployed outdoors.

Note that the controlled-setting results represent the end-point of a considerable experimental exploration. Formulating the CFD model that ultimately generated the comparison shown in Figure 7 and Table 1 required a number of design choices typical of CFD model construction. That is, the process of formulating a CFD model is neither “turn-key” nor “black-box”. By working with a scaled-down apparatus, we were able to develop and validate a model through repeated experiments that were not possible in an uncontrolled setting.

From the data, however, it is clear that the eventual model we developed is within statistical tolerance for the sensors we deployed in the small-scale, controlled setting. It is this model that we used with historical data to validate the results for the full-scale structure.

### 3.2. Back-Testing Results for Commercial-Scale CUPS

For validation in the full-scale CUPS structure, we selected a 25 min period from the historical dataset which has a stable average wind speed and direction. We define stable as varying less than 3 m/s between subsequent measurements and within 5 m/s of the period average. In addition, we only considered candidate periods with less than 20 degrees variance in wind direction, between measurements. We chose these parameters because they identified a period of time in the historical data archive during which the environmental conditions in and around the LREC CUPS were most similar to the conditions we were able to maintain in the controlled setting. That is, we identified in the historical archive of sensor data from the commercial-scale CUPS a period of time during which the inter-measurement difference was less than 3 m/s and all measurements were within 5 m/s of the average. During this period, we also needed the wind direction to be constant (as in the controlled setting) and from the north (to align the wind direction with the orientation of the CUPS and the sensors within it). Finally, we wished to use at least 5 measurement values to compute measurement confidence intervals. The smallest variation in wind direction over a 5 measurement period (25 min) when the wind was from the north, the inter-measurement differences were less than 3 m/s and the average was 5 m/s was 20 degrees. In addition, because of the limitations of cup anemometers, all wind speeds are measured only horizontally.

We built the OpenFOAM model in the same way as we did for the small-scale test structure. We use the dimensions of the actual CUPS structure and, again, choose the grid locations (after adaptive mesh refinement) closest to the anemometers in 3 dimensions.

**Figure 8:**
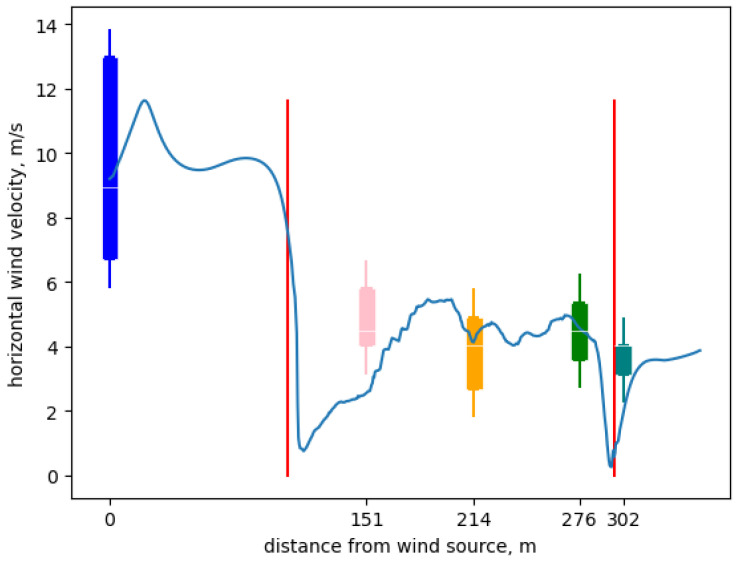
CUPS structure model back-test results. The blue line shows model prediction, the red lines depict the edges of the structure and the boxplots show sensor measurements.

Figure 8 shows the results of an example backtest for the 25 min period between 16:31 and 16:56 on 22 June 2022. We parameterized the initial conditions for the model using the wind speed measured at *north out* at the beginning of the time period. In the figure, the box-and-whiskers plots show the average, 0.95 quantiles, and the extrema for the measurement values taken from each location. The continuous blue line shows the modeled prediction converged to an average value for the horizontal path within the simulation that most nearly intersects the anemometer positions in three dimensions (i.e., the location within the simulation grid closest to each sensor). The distances along the *x*-axis are the horizontal distances from the *north out* sensor in meters. The red vertical lines identify the boundaries of the maximal rectangular volume within the CUPS structure.

Table 2 compares the average measurements for the five different anemometer locations to the corresponding average modeled values that were provided by the CFD computation. Note that for the historical data from the commercial-scale CUPS, the horizontal wind speed measurements were 1 min averages reported every 5 min. The units for the average measurements, the standard deviations, and the modeled values are all meters per second. Column 4 gives the two-sided 0.95 confidence interval on the average measurement given in column 2.

In this case, we compute each average from 5 measurements taken 5 min apart. The *t*-statistic for the 0.95 two-sided confidence bounds on these averages is 2.776 with 4 degrees of freedom. Note that only the modeled value for *south out* (boldfaced) falls outside its corresponding confidence interval. We believe that this the discrepancy is due to turbulence effects (which we do not model) caused by a growing block located immediately outside the CUPS on its south side next to the *south out* sensor. Like the data taken from the controlled setting, these data indicate that the interior modeled values are within the confidence intervals surrounding the corresponding average measurements under standard assumptions about the measurement uncertainty.

Given the resolution of the measurement data, this comparison is quite good. Taken with the data shown in Table 1, the evidence is that the OpenFOAM model is accurate to within the statistical tolerances of the measurement data in both the controlled setting and the in situ full-scale setting. Further the anemometer accuracy for the AcuRite sensors in (*middle in*, *south in*, and *south out*) as ±1.87 m/s and for the Davis Instruments sensors (*north out* and *north in*) as ±0.89 m/s according to their respective manufacturers. Thus, confidence bounds on the average measurements are likely wider than those given by a *t*-statistic [62]. At some small level of measurement uncertainty, the model and the measurements must differ, but determining this difference would require significantly more sophisticated and expensive anemometers than is practical for this setting.

## 4. Discussion

We performed two separate model validations of the same CFD model—one using a controlled “mock-up” of the commercial structure and the other using the structure itself. Each is intended to provide a different level of assurance that the CFD model represents airflow within the CUPS accurately. Our expectation was that the model would register with measurement more closely in a small-scale, controlled setting than in the field with the commercial structure. In the controlled setting, we were able to vary a single parameter of interest (the external wind vector) while holding all other interacting parameters relatively constant. We also used more accurate (but less durable) anemometers in the controlled setting to try and minimize the effects of measurement uncertainty on model validation. These results, summarized in Figure 7, provide confidence that the model correctly represents airflow through the structure at the meter scale.

We also wanted to understand the degree to which model accuracy was attenuated (versus the results shown in Figure 7) by the additional factors we could not control for the commercial-scale CUPS at LREC and by physical scale. Figure 8 shows that the CFD model’s accuracy is decreased relative to the controlled setting. However, Table 1 and Table 2 both indicate that the modeled values fall within the confidence intervals for the corresponding measurements in each setting. Thus, in the experiments using the commercial-scale CUPS and back-testing, it is possible to treat the effects of uncontrolled factors and scale as statistical measurement uncertainty for the purposes of evaluating model accuracy.

While our validation results are promising, it is important to acknowledge several limitations of the current study to make space for future work. By addressing the challenges that remain, we aim to develop a robust and reliable tool that can support decision-making in agriculture, ultimately leading to more sustainable and productive farming practices.

### 4.1. Validation vs. Application

In this study, we have focused on validating our proposed model rather than directly implementing it as part of an agricultural application. We made this decision for two reasons. First, we were unsure of the extent to which the modeling technique would be “successful” in terms of its accuracy and we did not want to build an application without first understanding how well the model would perform. Secondly, our anecdotal experience in fielding IoT systems for agriculture led us to believe that no application we developed based on such a model would be considered trustworthy without this validation.

Another important aspect of this work is that we chose a validation approach based on what we anticipated would be the ultimate requirements for the model, namely that it was able to predict the final conditions in the structure based on conditions outside the structure. That is, in all validation experiments, we used the measured airflow outside the structure (upwind) as the initial inputs in the model, and compared the modeled values to measurements taken from within the structure.

Our view, in this regard, was that this approach was the most practical validation since many growing regions are already instrumented with local weather stations. However, other validations are possible. For example, it is possible to parameterize the model with inputs from all but one of the sensors (not just the windward outside sensor). If our results had not indicated that the modeled results were within the bounds of statistical uncertainty, we would have conducted this validation. The consequence, however, would be that modeling a CUPS would depend critically on an array of sensors inside and outside the structure and not just sensors of external conditions.

The number of external sensors that will ultimately be necessary is an open question. Based on the results of this study, we believe that an upwind external sensor is sufficient. This result argues for at least 4 such sensors—one located outside each of the four sides of the structure—in a practical setting with the incident wind computed from two of the sensors. However, nearby structures and growing blocks might create external turbulence effects that we have not modeled or validated.

### 4.2. Real-Time Processing and Computational Demands

Our model is not yet capable of real-time processing, which is a critical requirement for future work. Our work does illustrate the baseline case of using a steady-state model and a single, relatively inexpensive, single CPU as a way of calibrating the degree of optimization that will be necessary to achieve real-time response. There are a few approaches to improving the wall-clock latency associated with computing the model results. Certainly, a distributed system with more powerful computing elements, including GPUs, is an appropriate platform. However, OpenFOAM does not support this deployment scenario as part of its freely available, open-source distribution. Commercially available CFD solvers do make these deployment options available. Thus, another open question concerns the dollar cost, in terms of the hardware and software resources, that will be necessary to achieve real-time or near real-time computing times.

One way to address this question is to employ currently supported parallel computing techniques such as the Message Passing Interface (MPI) could allow the model to split tasks across multiple processors and reduce overall computation time. Typical CFD speedups, however, indicate that this approach alone will be insufficient.

Another optimization approach is based on the observation that the main contribution to the computation time comes from detail-oriented computational meshing with relatively small cells. We plan to work on improving the meshing balance between small cells in the areas of high importance and large cells in the less significant areas, to lower the computational cost without significant loss of accuracy. Finally, we plan to investigate how using the results of prediction from OpenFOAM that have proven to be accurate in this work as training data for a model that require more initial data but process the calculation significantly faster, such as deep learning models.

### 4.3. Adding More Target Parameters

One of the most important advantages of the model we chose is that it is relatively easy to add other target parameters. It is important that the model is versatile and able to accommodate many requirements that agriculture imposes. Adding another parameter, such as temperature, requires only temperature input and temperature interactions with the existing 3D model rigid bodies. As a direct example of the next step in this area, we can add temperature since thermal spread equations can be a part of the current solver.

We believe that this extensibility is a feature of our approach. For example, heat transfer (which the current model computes but which we have not validated) will be important to applications such as frost prevention and irrigation scheduling. Validation of the model accuracy for these additional target parameters will likely require additional sensing infrastructure. However, the results of this study indicate that good model accuracy based on the physics of fluid flow is possible for these parameters as well.

## 5. Conclusions

With this paper, we present a novel advance in smart agriculture that provides climate modeling and prediction for Citrus Under Protective Screens (CUPS). CUPS structures are used in citrus production in Florida and elsewhere to exclude large insects (including the vector that causes citrus greening). However, screen structures also change the climate conditions (airflow, temperature, humidity, solar radiation, etc.) inside the CUPS, requiring that adjustments be made to farming practices (irrigation, spray applications, disease control, etc.). The goal of our research is to develop techniques that enable us to better understand the impacts of using CUPS for growing citrus in California in order to expedite adoption of CUPS and to encourage the development of smart agriculture solutions for CUPS structures.

In this paper, we develop and describe a computational fluid dynamics (CFD) model to capture, simulate, and predict the airflow patterns inside CUPS (from the airflow measurements outside of CUPS). Accurate predictions of airflow are key for informing application practices of sprays (e.g., pesticide) and water (for irrigation and frost mitigation) inside CUPS. We validate our model using both a scaled-down and commercial-scale CUPS deployment. We use an IoT deployment of devices and sensors to perform this validation. We find that our model is sufficiently accurate in both cases, suggesting that it has the potential to enhance the next generation in decision support and intelligent automation for CUPS growers.

## Figures and Tables

**Table 1:** Controlled test structure measurements.

Loc	Measurement (m/s)	Model Avg (m/s)	Conf. Int.
Loc1	avg: 2.58 sd: 0.20	2.65	(2.37, 2.79)
Loc2	avg: 1.78 sd: 0.14	1.87	(1.64, 1.94)
Loc3	avg: 1.50 sd: 0.11	1.68	(1.37, 1.61)
Loc4	avg: 1.29 sd: n/a	**1.24**	n/a

**Table 2:** Average CUPS modeled values compared to corresponding average measurements.

Location	Measurement (m/s)	Model Average (m/s)	Conf. Int.
*north out*	avg: 10.2 sd: 2.37	9.2	(7.27, 13.1)
*north in*	avg: 4.91 sd: 1.41	2.74	(3.17, 6.65)
*middle in*	avg: 4.73 sd: 1.56	4.16	(2.80, 6.66)
*south in*	avg: 4.91 sd: 0.83	4.95	(3.89, 5.93)
*south out*	avg: 3.66 sd: 0.49	**2.47**	(3.06, 4.26)

## Data Availability

The data from this study is available from https://github.com/MAYHEM-Lab/public-datasets (accessed on 20 September 2024) in folder CUPS-model-Aug2024.

## Abbreviations

The following abbreviations are used in this manuscript:

CUPS	Citrus Under Protective Screen
CFD	Computational Fluid Dynamics
DF	Darcy–Forchheimer
HLB	Huanglongbing disease (also known as citrus greening disease)
CPU	Central Processing Unit
GPU	Graphics Processing Unit
UV	Ultraviolet
3D	Three Dimensional
m/s	Meters Per Second
LCD	Liquid Crystal Display
LREC	Lindcove Research and Extension Center

## References

[1] National Agricultural Statistics Service (2023). Citrus Fruits 2023 Summary. https://downloads.usda.library.cornell.edu/usda-esmis/files/j9602060k/4742bs21j/3n205h50s/cfrt0923.pdf.

[2] Hall D.G., Richardson M.L., Ammar E.D., Halbert S.E. (2013). Asian citrus psyllid, D iaphorina citri, vector of citrus huanglongbing disease. Entomol. Exp. Appl..

[3] Munch D. (2013). U.S. Citrus Production—An Uphill Battle to Survive.

[4] Hodges A., Spreen T. (2012). Economic impacts of citrus greening (HLB) in Florida, 2006/07-2010/11. Electronic Data Information Source (EDIS) FE903.

[5] Agriculture U., Resources N. (2024). Distribution of ACP in California. https://ucanr.edu/sites/ACP/Distribution_of_ACP_in_California/.

[6] Citrus Pest & Disease Prevention Program (2024). Huanglongbing Quarantine. https://californiacitrusthreat.org/pest-disease/huanglongbing-quarantine/.

[7] Schumann A., Waldo L., Mariner N., Ebert T. (2020). Five years of fresh fruit production in CUPS. Citrus Ind. Mag..

[8] Ebert T., Schumann A., Waldo L., Stanton D., Rolshausen P. (2021). A Close-Up Look at Screens for Excluding Asian Citrus Psyllids.

[9] Schumann A.W., Singerman A., Ritenour M., Qureshi J., Alferez F. (2024). 2024–2025 Florida Citrus Production Guide: Citrus under Protective Screen (CUPS) Production Systems.

[10] Singerman A., Schumann A. (2023). Return-on-Investment Potential of Citrus under Protective Screens (CUPS). Citrus Industry White Paper. https://crec.ifas.ufl.edu/media/crecifasufledu/economics/docs/CUPS_20230825.pdf.

[11] Agehara S., Vallad G., Torres-Quezada E. (2012). Protected Culture for Vegetable and Small Fruit Crops: Types of Structures. Electronic Data Information Source (EDIS) HS1224.

[12] University of California Department of Agriculture and Natural Resources Welcome—lrec.ucanr.edu. https://lrec.ucanr.edu/.

[13] Cao G., Awbi H., Yao R., Fan Y., Sirén K., Kosonen R., Zhang J.J. (2014). A review of the performance of different ventilation and airflow distribution systems in buildings. Build. Environ..

[14] Mirzaie M., Lakzian E., Khan A., Warkiani M.E., Mahian O., Ahmadi G. (2021). COVID-19 spread in a classroom equipped with partition—A CFD approach. J. Hazard. Mater..

[15] Nielsen P.V. (2004). Computational fluid dynamics and room air movement. Indoor Air.

[16] Slingo J., Palmer T. (2011). Uncertainty in weather and climate prediction. Philos. Trans. R. Soc. Math. Phys. Eng. Sci..

[17] Lawan S., Abidin W., Chai W., Baharun A., Masri T. (2014). Different models of wind speed prediction; a comprehensive review. Int. J. Sci. Eng. Res..

[18] OpenFOAM—Openfoam.com. https://www.openfoam.com/.

[19] Ferrarezi R.S., Qureshi J.A., Wright A.L., Ritenour M.A., Macan N.P.F. (2019). Citrus Production Under Screen as a Strategy to Protect Grapefruit Trees From Huanglongbing Disease. Front. Plant Sci..

[20] AcuRite Iris Personal Weather Stations—Personal Weather Stations—Shop for Weather—Shop All—Acurite.com. https://www.acurite.com/shop-all/weather-instruments/weather-stations/iris.html?srsltid=AfmBOoqH_TRDRqAjbuMtQCQNoWvkgo7ppGCLVi24djVjcpl0-DUMOfSu.

[21] Vantage Pro2—Davisinstruments.com. https://www.davisinstruments.com/collections/vantage-pro2?srsltid=AfmBOoqaY0U6K2F-3xuWTlRZ-3yE6LfvYtnmDXRyaIeQimtSL6bPQqQ.

[22] Martin-Clouaire R. (2017). Modelling Operational Decision-Making in Agriculture. Agric. Sci..

[23] Ascough J.C., Shaffer M.J., McMaster G.S., Deer-Ascough L. The Great Plains Framework for Agricultural Resource Management (GPFARM): A decision support system for whole farm/ranch strategic planning. Proceedings of the Sustaining the Global Farm (2001): Selected papers from the 1999 International Soil Conservation Organization Meeting.

[24] Glen J.J. (1987). Feature Article—Mathematical Models in Farm Planning: A Survey. Oper. Res..

[25] Jayaraman P.P., Yavari A., Georgakopoulos D., Morshed A., Zaslavsky A. (2016). Internet of things platform for smart farming: Experiences and lessons learnt. Sensors.

[26] Maddikunta P.K.R., Hakak S., Alazab M., Bhattacharya S., Gadekallu T.R., Khan W.Z., Pham Q.V. (2021). Unmanned aerial vehicles in smart agriculture: Applications, requirements, and challenges. IEEE Sens. J..

[27] Gonzalez-De-Santos P., Fernández R., Sepúlveda D., Navas E., Armada M. (2020). Unmanned ground vehicles for smart farms. Agron. Clim. Chang. Food Secur..

[28] Paul K., Chatterjee S.S., Pai P., Varshney A., Juikar S., Prasad V., Bhadra B., Dasgupta S. (2022). Viable smart sensors and their application in data driven agriculture. Comput. Electron. Agric..

[29] Golubovic N., Krintz C., Wolski R., Sethuramasamyraja B., Liu B. (2018). A Scalable System for Executing and Scoring K-Means Clustering Techniques and Its Impact on Applications in Agriculture. Int. J. Big Data Intell..

[30] Adamides G., Kalatzis N., Stylianou A., Marianos N., Chatzipapadopoulos F., Giannakopoulou M., Papadavid G., Vassiliou V., Neocleous D. (2020). Smart farming techniques for climate change adaptation in Cyprus. Atmosphere.

[31] Park J., Moon A., Lee E., Kim S. (2021). Understanding IoT climate data based predictive model for outdoor smart farm. Proceedings of the 2021 International Conference on Information and Communication Technology Convergence (ICTC).

[32] Frandsen S., Barthelmie R., Pryor S., Rathmann O., Larsen S., Højstrup J., Thøgersen M. (2006). Analytical modelling of wind speed deficit in large offshore wind farms. Wind. Energy Int. J. Prog. Appl. Wind. Power Convers. Technol..

[33] Santamaría-Bonfil G., Reyes-Ballesteros A., Gershenson C. (2016). Wind speed forecasting for wind farms: A method based on support vector regression. Renew. Energy.

[34] Devis A., Van Lipzig N.P., Demuzere M. (2018). Should future wind speed changes be taken into account in wind farm development?. Environ. Res. Lett..

[35] Li G., Shi J. (2010). On comparing three artificial neural networks for wind speed forecasting. Appl. Energy.

[36] Mohamadi F., Fazeli A. (2022). A review on applications of CFD modeling in COVID-19 pandemic. Arch. Comput. Methods Eng..

[37] Bhattacharyya S., Dey K., Paul A.R., Biswas R. (2020). A novel CFD analysis to minimize the spread of COVID-19 virus in hospital isolation room. Chaos Solitons Fractals.

[38] Alrebi O.F., Obeidat B., Abdallah I.A., Darwish E.F., Amhamed A. (2022). Airflow dynamics in an emergency department: A CFD simulation study to analyse COVID-19 dispersion. Alex. Eng. J..

[39] Schindler D., Bauhus J., Mayer H. (2012). Wind effects on trees. Eur. J. For. Res..

[40] Endalew A.M., Hertog M., Delele M., Baetens K., Persoons T., Baelmans M., Ramon H., Nicolaï B., Verboven P. (2009). CFD modelling and wind tunnel validation of airflow through plant canopies using 3D canopy architecture. Int. J. Heat Fluid Flow.

[41] Sellier D., Brunet Y., Fourcaud T. (2008). A numerical model of tree aerodynamic response to a turbulent airflow. Forestry.

[42] Li R., Zhao Y., Wang L.L., Niu J., Shi X., Gao N. (2024). Numerical investigation of the blockage effect of trees on airflow distributions in a wind tunnel. Build. Environ..

[43] Huang J., Hao T., Wang Y., Jones P. (2022). A street-scale simulation model for the cooling performance of urban greenery: Evidence from a high-density city. Sustain. Cities Soc..

[44] van Druenen T., Van Hooff T., Montazeri H., Blocken B. (2019). CFD evaluation of building geometry modifications to reduce pedestrian-level wind speed. Build. Environ..

[45] Talaviya T., Shah D., Patel N., Yagnik H., Shah M. (2020). Implementation of artificial intelligence in agriculture for optimisation of irrigation and application of pesticides and herbicides. Artif. Intell. Agric..

[46] Valdivia-Bautista S.M., Domínguez-Navarro J.A., Pérez-Cisneros M., Vega-Gómez C.J., Castillo-Téllez B. (2023). Artificial Intelligence in Wind Speed Forecasting: A Review. Energies.

[47] Banks D.L., Fienberg S.E., Meyers R.A. (2003). Data Mining, Statistics. Encyclopedia of Physical Science and Technology.

[48] Bear J. (2013). Dynamics of Fluids in Porous Media.

[49] Xiong Q., Baychev T.G., Jivkov A.P. (2016). Review of pore network modelling of porous media: Experimental characterisations, network constructions and applications to reactive transport. J. Contam. Hydrol..

[50] Alazmi B., Vafai K. (2000). Analysis of variants within the porous media transport models. J. Heat Mass Transf..

[51] Hematpur H., Mahmood S.M., Nasr N.H., Elraies K.A. (2018). Foam flow in porous media: Concepts, models and challenges. J. Nat. Gas Sci. Eng..

[52] Coulaud O., Morel P., Caltagirone J. (1988). Numerical modelling of nonlinear effects in laminar flow through a porous medium. J. Fluid Mech..

[53] Nield D.A., Bejan A. (2006). Convection in Porous Media.

[54] Foundation B. blender.org—Home of the Blender project—Free and Open 3D Creation Software—blender.org. https://www.blender.org/.

[55] OpenFOAM: API Guide: Applications/Utilities/Surface/SurfaceFeatureExtract/SurfaceFeatureExtract. C File Reference—Openfoam.com. https://www.openfoam.com/documentation/guides/latest/api/surfaceFeatureExtract_8C.html.

[56] OpenFOAM: Manual Pages: PorousSimpleFoam(1)—Openfoam.com. https://www.openfoam.com/documentation/guides/latest/man/porousSimpleFoam.html.

[57] OpenFOAM Documentation—Reynolds Averaged Simulation (RAS)—doc.openfoam.com. https://doc.openfoam.com/2306/tools/processing/models/turbulence/ras/.

[58] ParaView—Open-Source, Multi-Platform Data Analysis and Visualization Application—Paraview.org. https://www.paraview.org/.

[59] UT363/UT363BT Mini Anemometers—UNI-T Meters|Test & Measurement Tools and Solutions—Meters.Uni-Trend.com. https://meters.uni-trend.com/product/ut363-ut363bt/.

[60] SnappyHexMesh-OpenFOAMWiki—Openfoamwiki.net. https://openfoamwiki.net/index.php/SnappyHexMesh.

[61] Farrance I., Frenkel R. (2012). Uncertainty of measurement: A review of the rules for calculating uncertainty components through functional relationships. Clin. Biochem. Rev..

[62] Snow J.M., Stuckman B.E., Usher J.S. (1993). Accuracy requirements for measurement systems with uniform and normal errors. Nav. Eng. J..

